# Urocortin 3 Marks Mature Human Primary and Embryonic Stem Cell-Derived Pancreatic Alpha and Beta Cells

**DOI:** 10.1371/journal.pone.0052181

**Published:** 2012-12-14

**Authors:** Talitha van der Meulen, Ruiyu Xie, Olivia G. Kelly, Wylie W. Vale, Maike Sander, Mark O. Huising

**Affiliations:** 1 Peptide Biology Laboratories, The Salk Institute for Biological Studies, La Jolla, California, United States of America; 2 Department of Pediatrics and Cellular and Molecular Medicine, University of California San Diego, La Jolla, California, United States of America; 3 ViaCyte, Inc., San Diego, California, United States of America; 4 Salk Center for Nutritional Genomics, The Salk Institute for Biological Studies, La Jolla, California, United States of America; Joslin Diabetes Center, Harvard Medical School, United States of America

## Abstract

The peptide hormone Urocortin 3 (Ucn 3) is abundantly and exclusively expressed in mouse pancreatic beta cells where it regulates insulin secretion. Here we demonstrate that Ucn 3 first appears at embryonic day (E) 17.5 and, from approximately postnatal day (p) 7 and onwards throughout adult life, becomes a unifying and exclusive feature of mouse beta cells. These observations identify Ucn 3 as a potential beta cell maturation marker. To determine whether Ucn 3 is similarly restricted to beta cells in humans, we conducted comprehensive immunohistochemistry and gene expression experiments on macaque and human pancreas and sorted primary human islet cells. This revealed that Ucn 3 is not restricted to the beta cell lineage in primates, but is also expressed in alpha cells. To substantiate these findings, we analyzed human embryonic stem cell (hESC)-derived pancreatic endoderm that differentiates into mature endocrine cells upon engraftment in mice. Ucn 3 expression in hESC-derived grafts increased robustly upon differentiation into mature endocrine cells and localized to both alpha and beta cells. Collectively, these observations confirm that Ucn 3 is expressed in adult beta cells in both mouse and human and appears late in beta cell differentiation. Expression of Pdx1, Nkx6.1 and PC1/3 in hESC-derived Ucn 3^+^ beta cells supports this. However, the expression of Ucn 3 in primary and hESC-derived alpha cells demonstrates that human Ucn 3 is not exclusive to the beta cell lineage but is a general marker for both the alpha and beta cell lineages. Ucn 3^+^ hESC-derived alpha cells do not express Nkx6.1, Pdx1 or PC1/3 in agreement with the presence of a separate population of Ucn 3^+^ alpha cells. Our study highlights important species differences in Ucn 3 expression, which have implications for its utility as a marker to identify mature beta cells in (re)programming strategies.

## Introduction

Urocortin 3 (Ucn 3) is a peptide hormone that belongs to the corticotropin-releasing factor (CRF) subfamily of peptide hormone, which also includes Ucn 1 and −2 [1], [2], [3]. Each peptide activates at least one of two closely related CRF receptors, CRFR1 and CRFR2, which belong the class B family of G protein-coupled receptors. Ucn 3 is abundantly and exclusively expressed in beta cells of the mouse pancreas [4] where it is required for full glucose- and incretin-stimulated insulin secretion [5]. Ucn 3 secretion from the beta cell is glucose-dependent and involves the ATP-sensitive potassium (K_ATP_) channel [5]. These islet-autonomous actions of Ucn 3 suggest the local presence of cognate receptors, which we confirmed by demonstrating expression of the alpha isoform of the CRFR2 receptor in MIN6 insulinoma cells and primary rodent and human islets [6].

Great progress has been made over the last decade in the ability to promote the differentiation of hESCs towards beta cells. Our increased understanding of the complex sequence of events that is required to drive beta cell differentiation culminated in detailed *in vitro* differentiation protocols [7], [8], [9]. While these protocols are effective in driving the differentiation from hESCs to pancreatic endoderm and endocrine progenitor cells *in vitro*, the resulting cells can only mature into glucose responsive functional beta cells when implanted into rodent recipients [9], [10], [11]. By contrast, endocrine differentiation of hESC-derived pancreatic endoderm *in vitro* produces insulin^+^ cells that co-express multiple endocrine hormones and fail to secrete insulin in a regulated manner [12]. Therefore, markers for mature functional beta cells that can be used to screen for compounds promoting beta cell differentiation *in vitro* are of considerable interest to the field of diabetes research. Similarly, strategies that seek to generate beta cells through transdifferentiation from diverse sources, such as non-beta endocrine, acinar, liver, and biliary epithelial cells [13], [14], [15], [16], [17], [18] would benefit from a maturation marker to help distinguish mature glucose-responsive and functional beta cells from immature insulin^+^ cells. Here we describe that Ucn 3 marks beta cells in rodents relatively late in development and is expressed in hESC-derived Pdx1^+^, Nkx6.1^+^ and PC1/3^+^ mature beta cells after engraftment *in vivo*. However, we also find that in primates Ucn 3 expression is not restricted to the beta cell lineage, but marks alpha cells as well. While these observations identify Ucn 3 expression as a feature of mature beta cells in rodents, they suggest that in humans, Ucn 3 is instead a general maturation marker of alpha and beta cells.

## Results

### Ucn 3 is expressed in mouse beta cells starting at E17.5

**Figure 1:**
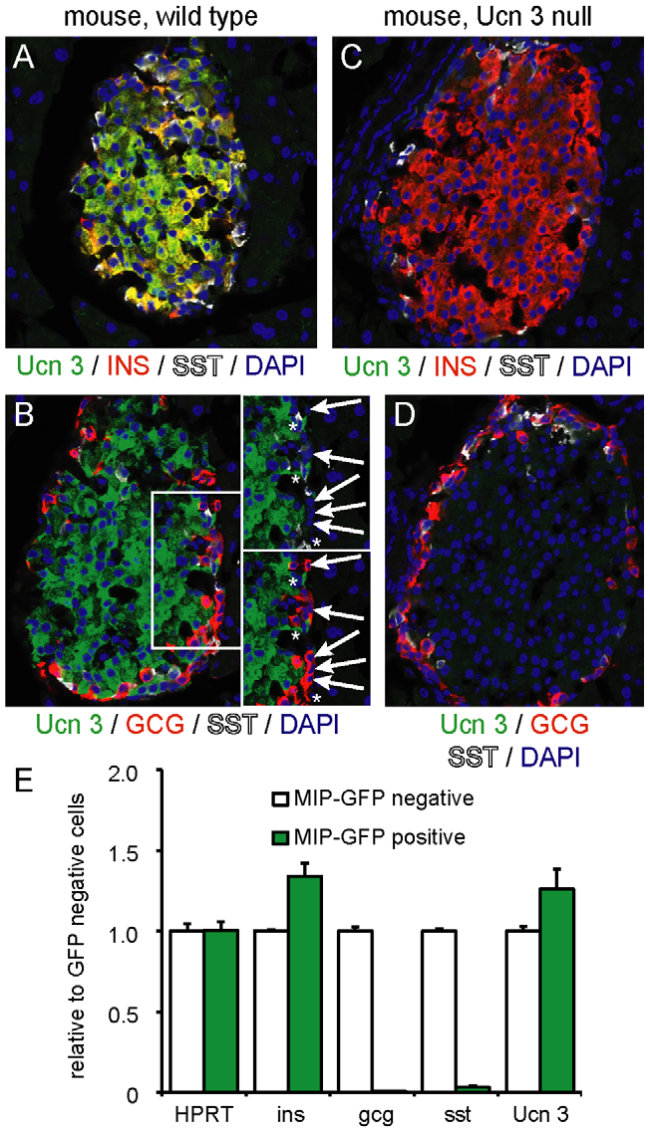
Ucn 3 expression in adult mouse islets is restricted to beta cells. Ucn 3 immunoreactivity overlaps completely with insulin (A) and not glucagon (B). Staining of the pancreas of a Ucn 3 null littermate demonstrates complete abrogation of Ucn 3 immunoreactivity to confirm the specificity of our antiserum (C, D). Details of panel C without glucagon (top) or somatostatin (bottom) channel reveals no Ucn 3 signal in alpha cells (arrows) or delta cells (asterisks). Expression profiling of dissociated FACS-sorted primary mouse beta cells from *MIP-GFP* transgenic mice demonstrates that purified beta cells have lost glucagon and somatostatin expression and are enriched for insulin and Ucn 3 expression (E). Note that both insulin and Ucn 3 expression remain present in the GFP-negative fraction due to the mosaic expression of the GFP reporter in this transgenic line.

We previously reported that Ucn 3 expression in the adult murine islet is exclusive to beta cells [4]. Here we confirmed and extended these findings by demonstrating that Ucn 3 fully overlapped with insulin in islets of adult wild type mice, but did not co-localize with glucagon or somatostatin (Fig. 1A, B). Ucn 3 immunoreactivity was abolished in islets of Ucn 3 null littermates, underscoring the specificity of our antiserum (Fig. 1C, D). Ucn 3 expression was retained in purified mouse primary beta cells obtained by FACS sorting dissociated islets from *MIP-GFP* reporter mice, further confirming the localization of Ucn 3 expression to beta cells (Fig. 1E), while expression of the alpha cell marker glucagon and the delta cell marker somatostatin is lost. Note that both insulin and Ucn 3 expression remain present in the GFP-negative fraction due to the mosaic expression of the GFP reporter in only approximately half of all beta cells of this transgenic line, as discussed elsewhere [19], [20].

**Figure 2:**
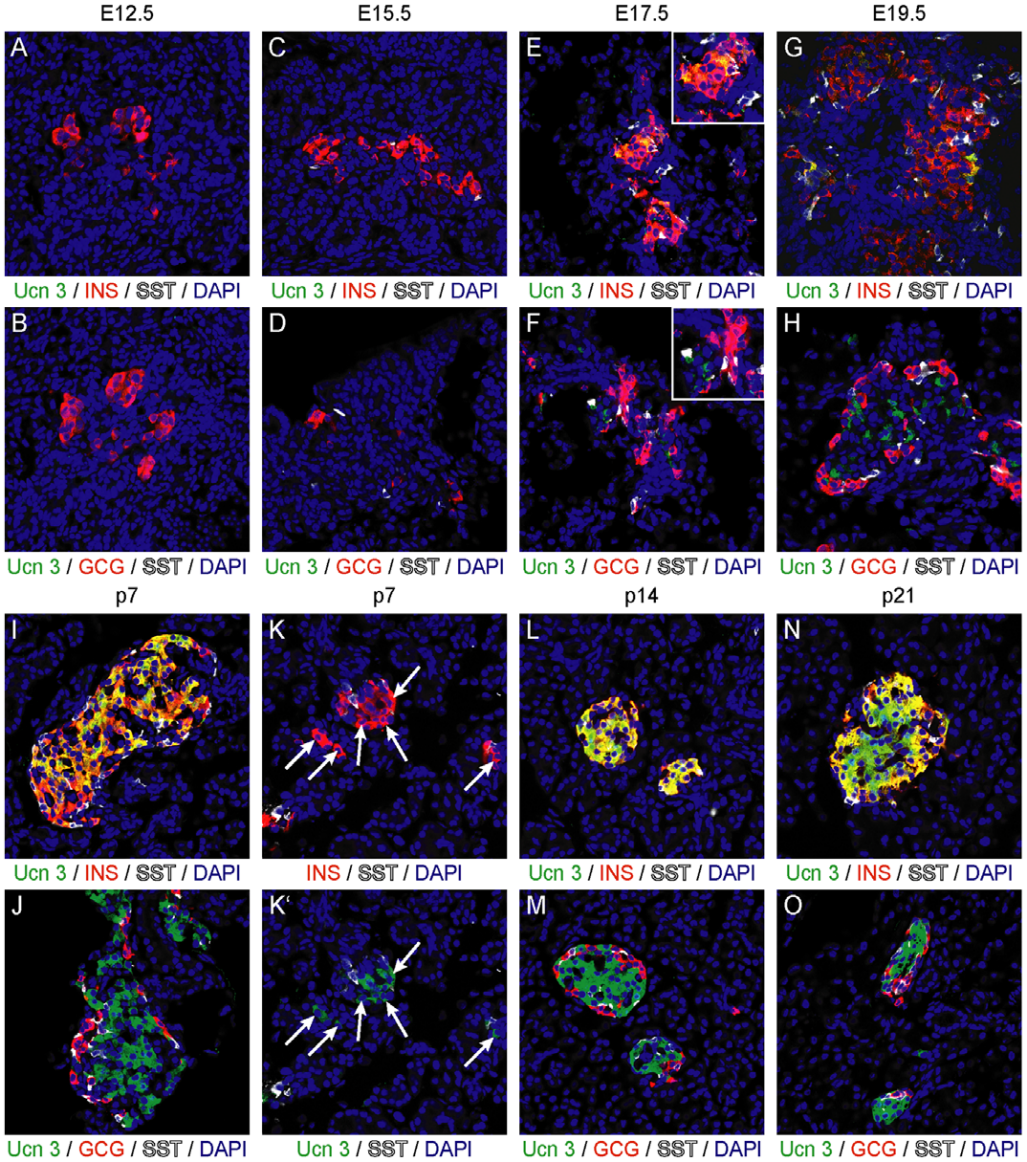
Ucn 3 expression trails the appearance of insulin in ontogeny. Ucn 3 expression, undetectable by immunohistochemistry at E12.5 (A, B) and E15.5 (C, D), first appears in a subset of beta cells around E17.5 of embryonic development (E, F). The fraction of Ucn 3^+^ beta cells increases perinatally (G, H) and by p7, the majority of beta cells (I, J), including those appearing as singlets in the acinar tissue (K) are clearly Ucn 3^+^. From p14 onwards, Ucn 3 expression is evident in all beta cells (L–O).

The first appearance of Ucn 3 in embryonic development and the extent of its overlap with insulin and other endocrine markers have not been comprehensively analyzed. We examined Ucn 3 expression by immunohistochemistry at different stages of embryonic development and early post-natal life. We observed Ucn 3 expression as early as E17.5 in a subset of insulin^+^ cells (Fig. 2E, F). No Ucn 3 immunoreactivity was observed at E12.5 or E15.5 (Fig. 2A–D). At E19.5, as islets are adopting their distinctive architecture, the number of Ucn 3^+^ beta cells increased (Fig. 2G, H) and by p7 the majority of insulin^+^ cells co-expressed Ucn 3 (Fig. 2I, J). This includes the insulin^+^ cells that appear in singlets or small clusters in the acinar tissue, although Ucn 3 staining in these cells was weaker compared to Ucn 3 expression within islets in the first week of life (Fig. 2K, K'). From p14 onwards, Ucn 3 and insulin expression completely overlapped (Fig. 2L–O). We did not observe co-localization of Ucn 3 with glucagon or somatostatin at any point during mouse embryonic development or postnatal life.

### Ucn 3 expression is a characteristic shared by primate alpha and beta cells

**Figure 3:**
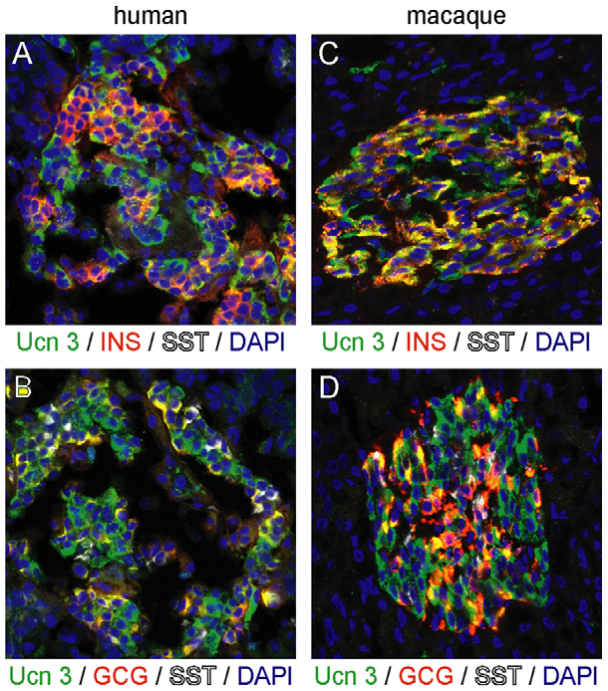
Ucn 3 expression in the primate pancreas is a feature of alpha and beta cells. Ucn 3 immunoreactivity co-localizes with insulin as well as glucagon in both human (A, B) and macaque (C, D) pancreas.

**Figure 4:**
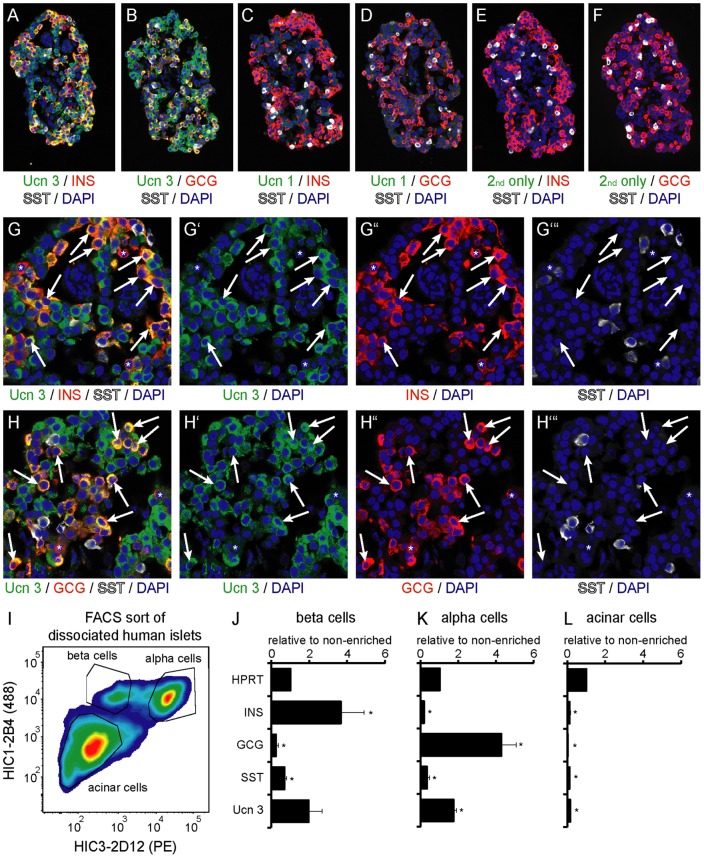
Ucn 3 is expressed in alpha and beta cells in freshly fixed human islets. Serial sections of the same human islet demonstrate that Ucn 3 expression co-localizes with insulin (A) and glucagon (B), but not somatostatin. The same islet shows no immunoreactivity for the related peptide Ucn 1 using an antiserum similar to the one used for the detection of Ucn 3 (C, D). Additional controls stained with secondary antibody only are negative as well (E, F). High power magnifications illustrate that the large majority of beta (G) and alpha (H) cells co-express Ucn 3 (arrows), although occasional insulin^+^ and glucagon^+^ cells can be found that do not stain for Ucn 3 (asterisks in G and H). Ucn 3 gene expression in FACS-purified dissociated human islet cells (I) from three individual donors confirmed that Ucn 3 is enriched in human beta (J) and alpha cells (K). The acinar cell fraction was depleted for the expression of all endocrine markers (L). Gene expression is normalized to HPRT and expressed relative to pre-sort levels. Error bars indicate standard errors, asterisks reflect significant differences at a p-value <0.05 as determined by t-test.

As species differences between primate and rodent islets are well documented [21], [22], [23], [24], we next assessed the expression pattern of Ucn 3 in human pancreas. In agreement with our previous observations in mouse, Ucn 3 immunoreactivity in human pancreas co-localized with insulin (Fig. 3A). However, while Ucn 3 expression in the mouse pancreas is restricted to the beta cell lineage, a sizeable fraction of Ucn 3^+^ cells in the human pancreas did not express insulin, but instead co-expressed glucagon (Fig. 3B). As the procurement process for human pancreas can pose a challenge to obtain pristine tissue quality, we obtained a pancreas specimen from a pig-tailed macaque (*Macaca nemestrina*), collected and fixed immediately post-mortem. Immunostaining of serial sections of macaque pancreas confirmed that Ucn 3 co-localizes with insulin (Fig. 3C) and glucagon (Fig. 3D) in islets. We also assessed Ucn 3 expression in human donor islets that were immersion fixed upon arrival. Again Ucn 3 expression in serial sections of the same islet was evident in the large majority of alpha and beta cells with no Ucn 3 expression in delta cells (Fig. 4A–B), findings that were confirmed using a second Ucn 3 antiserum (not shown). Controls stained for Ucn 1 (Fig. 4C, D), a peptide related to Ucn 3 that is not expressed in islets, or controls without primary antibody (Fig. 4E, F) did not show any staining, confirming the specificity of our Ucn 3 antiserum on the human material. At higher magnification, the colocalization of Ucn 3 with insulin and glucagon is evident in the large majority of beta and alpha cells, respectively, although there are occasional insulin^+^ and glucagon^+^ cells that do not co-express Ucn 3 (Fig. 4G, H).

To substantiate our findings with a method independent of the Ucn 3 antiserum, we conducted gene expression analysis on FACS sorted dissociated primary human islet cells. We collected FACS-purified human islet fractions from three individual donors, using an established method based on validated monoclonal antibodies against human islet cell surface markers [25]. Quantitative PCR analysis of these purified islet populations (Fig. 4I) demonstrated that the human beta cell fraction was enriched for insulin and Ucn 3 and depleted for glucagon expression, as expected (Fig. 4J). The human alpha cell fraction showed increased glucagon expression and was depleted for the expression of both insulin and somatostatin, confirming successful alpha cell enrichment. Ucn 3 expression was enriched along with glucagon in the human alpha cell population, providing independent confirmation that not only human beta cells but also alpha cells expressed Ucn 3 (Fig. 4K). The acinar cell fraction showed robust depletion for all endocrine markers (Fig. 4L).

### Ucn 3 expression is induced during maturation of hESC-derived alpha and beta cells

**Figure 5:**
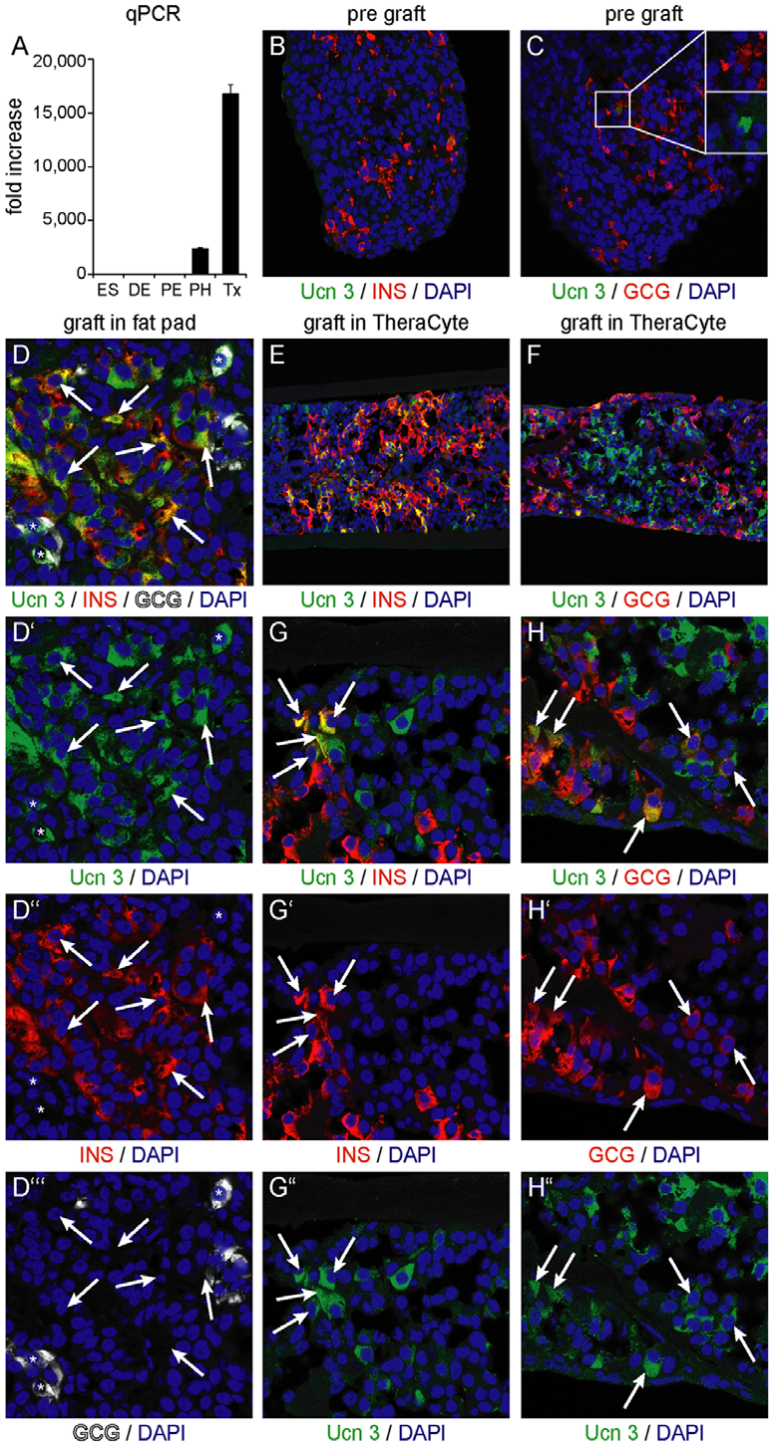
Expression of Ucn 3 in hESC-cell derived alpha and beta cells requires *in vivo* maturation. Quantitative PCR analysis of key differentiation stages towards the human beta cell lineage reveals no Ucn 3 expression at the embryonic stem cell (ES) or definitive endoderm (DE) stage. While pancreatic cultures enriched for pancreatic endoderm cells (PE) by CD142 did not express Ucn 3, polyhormonal cells (PH) enriched from the same cultures by CD200, started to express Ucn 3. Implants of hESC-derived pancreatic progenitors robustly express Ucn 3 after differentiation and maturation *in vivo* at 140 days post-transplant (Tx). Ucn 3 expression is measured by qRT-PCR, normalized to TATA-box binding protein (TBP) and plotted relative to ES cells (A). These observations are confirmed on *in vitro* differentiated hESC-derived pancreatic endoderm, which contains a minor fraction of polyhormonal cells that express insulin (B) and glucagon (C), but are mostly devoid of Ucn 3 immunoreactivity with the exception of faint Ucn 3 expression in the occasional glucagon^+^ cell (C, inset). Ucn 3 immunoreactivity is robustly upregulated 18 weeks following engraftment in the epidydimal fat pads of mice and demonstrates significant Ucn 3 colocalization with insulin^+^ beta cells and glucagon^+^ alpha cells (D). Ucn 3 immunoreactivity is robustly upregulated 140 days post implantation in TheraCyte encapsulation devices and co-localizes with both insulin (E) and glucagon (F). Heterogeneity exists among both alpha and beta cells regarding the expression of Ucn 3. Arrows indicate Ucn 3^+^ cells that co-express insulin (G) or glucagon (H).

A recent study identified Ucn 3 as a beta cell-specific maturation marker and suggested potential utility to distinguish immature from mature hESC-derived beta cells [26]. However, our data in macaque and human pancreas and human donor islets suggest that Ucn 3 expression in primates is not restricted to the beta cell lineage. Therefore, we evaluated Ucn 3 expression in hESCs in comparison to key intermediate differentiation stages towards the formation of hESC-derived mature endocrine cells. Expression profiles obtained by qRT-PCR revealed little expression of Ucn 3 mRNA in embryonic stem cells or definitive endoderm cultures. CD142-enriched pancreatic endoderm cells did not express Ucn 3, while CD200-enriched polyhormonal cells derived from the same pancreatic cultures [11] started to express Ucn 3. High expression of Ucn 3 was detected in grafts 140 days following implantation *in vivo* (Fig. 5A). These findings demonstrated robust induction of Ucn 3 following engraftment and maturation of hESC-derived pancreatic endoderm *in vivo*, but did not identify the cellular source of its expression. Therefore, we assessed Ucn 3 expression by immunohistochemistry following engraftment of hESC-derived pancreatic endoderm in recipient mice. Prior to transplant the hESC-derived pancreatic endoderm contained a minor fraction of insulin^+^ and glucagon^+^ cells, which are mostly polyhormonal cells that do not contribute to the formation of mature beta cells [11]. Ucn 3 was undetectable in the majority of these hormone^+^ cells (Fig. 5B, C), although faint Ucn 3 immunoreactivity was occasionally observed in glucagon^+^ cells (Fig. 5C, inset), in line with the start of Ucn 3 expression in polyhormonal cells by qRT-PCR (Fig. 5A). In close agreement to our qRT-PCR findings, Ucn 3 expression markedly increased to label 79% of beta cells and 68% of alpha cells (107 out of 136 beta cells and 102 out of 150 alpha cells, counted across 7 random fields of view) following grafting in the epidydimal fat pad of recipient mice for 128 days (Fig. 5D). Similar findings were obtained in grafts transplanted subcutaneously in a TheraCyte encapsulation device for 140 days (38%, or 331 out of 862 insulin^+^ cells and 45%, or 126 out of 278 glucagon^+^ cells counted across 18 random fields of view) (Fig. 5E–F). Only a small fraction of cells (<4% or 19 out of 476 Ucn 3^+^ cells) appeared to express Ucn 3 but not insulin or glucagon (not shown). The origins and significance of this small Ucn 3^+^ population that does not co-express insulin or glucagon is currently not known. Nevertheless, the large majority of Ucn 3^+^ cells expressed insulin (Fig. 5G) or glucagon (Fig. 5H), in agreement with our earlier findings demonstrating that Ucn 3 marked alpha as well as beta cells in human and macaque pancreas and sorted human islet cells.

**Figure 6:**
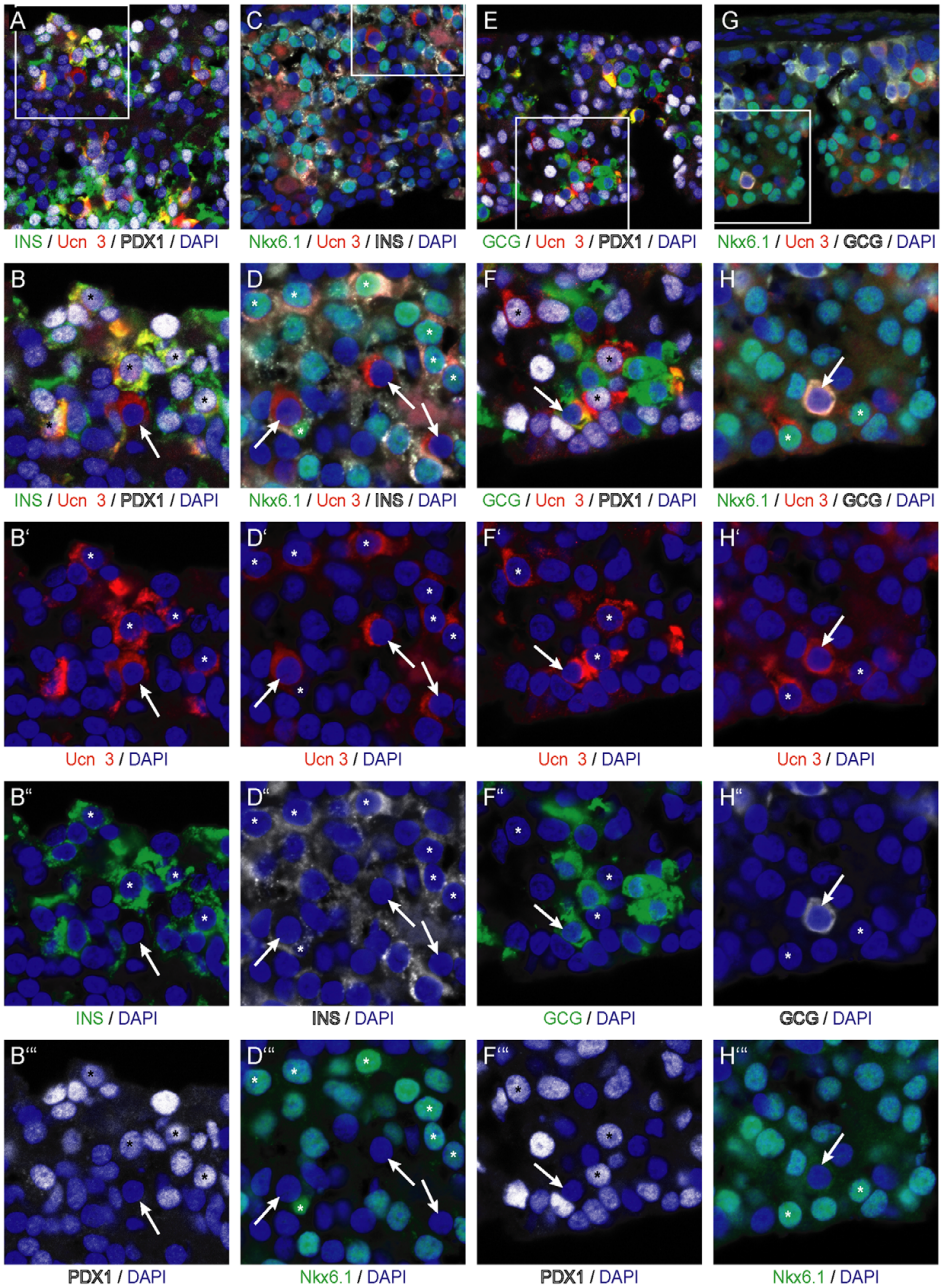
Ucn 3 expression co-localizes with Pdx1 and Nkx6.1 in beta cells but not in alpha cells. Ucn 3 in beta cells differentiated in implants of hESC-derived pancreatic endoderm cells co-labels with Pdx1 (A, B) or Nkx6.1 (C, D). Ucn 3 in glucagon^+^ alpha cells does not co-localize with Pdx1 (E, F) or Nkx6.1 (G, H). Arrows in all panels indicate Ucn 3^+^ alpha cells, whereas asterisks denote Ucn 3^+^ mature beta cells. Boxed areas in the first row of images are magnified in subsequent panels.

The observation that Ucn 3 expression in grafts of hESC-derived pancreatic endoderm at the time of graft collection is restricted to a subset of beta cells is reminiscent of our findings during development, where Ucn 3 is not expressed in the majority of beta cells until p7 (Fig. 2) and indicates that Ucn 3 marks a more mature subset of beta cells, as has been previously suggested [26]. To substantiate, we co-labeled grafts of hESC-derived pancreatic endoderm following transplantation *in vivo* for Ucn 3, insulin and glucagon as well as Pdx1 and Nkx6.1. These experiments demonstrate that Ucn 3^+^ cells that co-express insulin, also express Pdx1 (Fig. 6A, B) or Nkx6.1 (Fig. 6C, D). This supports the identity of Ucn 3 as a maturity marker whose expression in the beta cell lineage follows that of Pdx1, Nkx6.1 and insulin. In contrast, Ucn 3 expression in alpha cells, identified by co-labeling with glucagon, does not overlap with Pdx1 (E, F) or Nkx6.1 (G, H), whose expression is lost from mature alpha cells.

**Figure 7:**
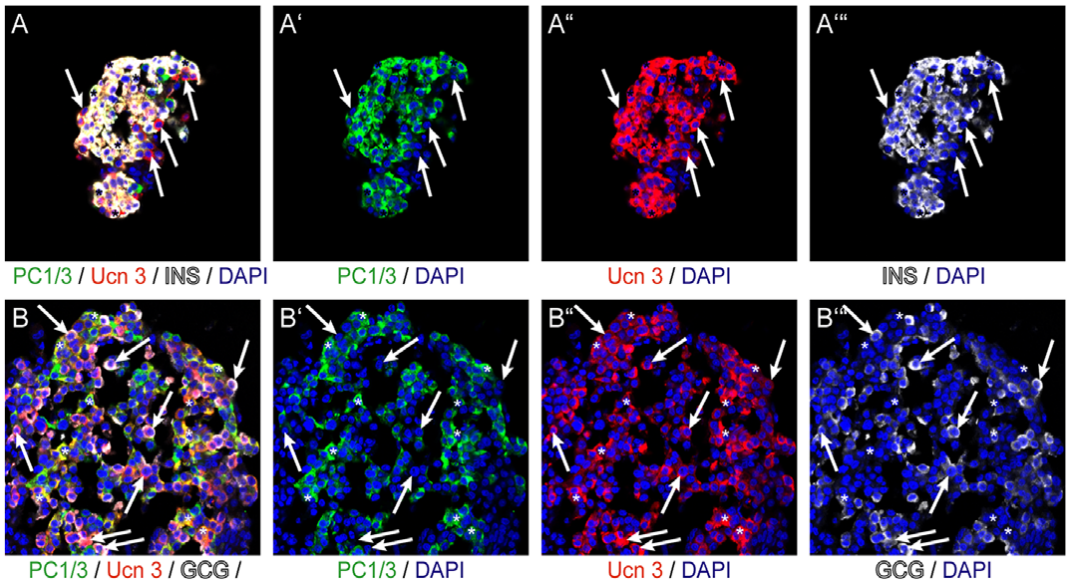
Ucn 3 expression in freshly fixed human islets co-localizes with PC1/3 in beta cells but not in alpha cells. Ucn 3 expression in human insulin+ beta cells coincides with PC1/3 (A), while Ucn 3+ glucagon+ alpha cells do not express PC1/3 (B). Arrows in all panels indicate Ucn 3^+^ alpha cells, whereas asterisks denote Ucn 3^+^ mature beta cells.

**Figure 8:**
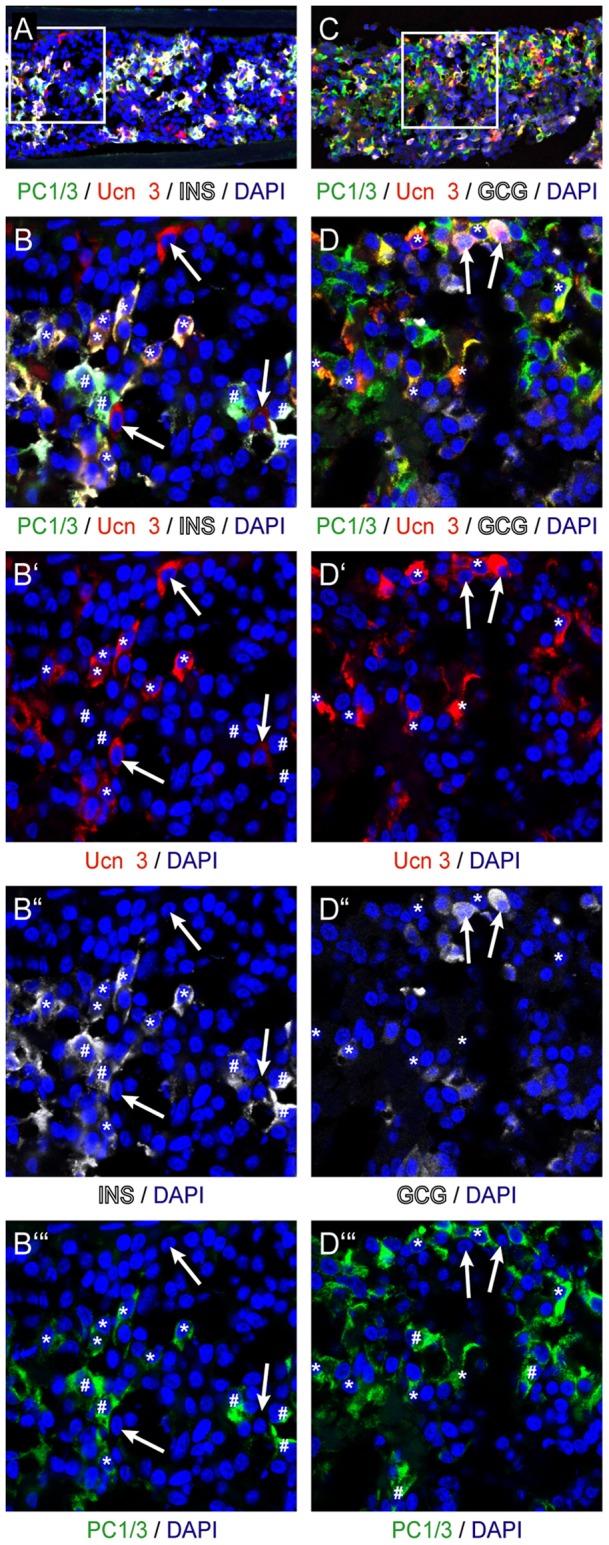
Ucn 3 expression in hESC-derived beta cells co-localizes with PC1/3. Ucn 3 in beta cells differentiated in implants of hESC-derived pancreatic endoderm cells co-labels with PC1/3 (A, B), while Ucn 3 in glucagon^+^ alpha cells does not (C, D). Arrows in all panels indicate Ucn 3^+^ alpha cells, whereas asterisks denote Ucn 3^+^ mature beta cells. Pound signs indicate insulin^+^, PC1/3^+^ Ucn 3^-^ immature beta cells. Boxed areas in the first row of images are magnified in subsequent panels.

While continued expression of Pdx1 and Nkx6.1 in beta cells is required for the transcriptional program that maintains beta cell identity [27], [28], they do not directly participate in the processing and secretion of insulin. Therefore, we colocalized Ucn 3 with PC1/3, which is a prohormone convertase required for pro-insulin to insulin processing [29]. In human islets, PC1/3 expression overlaps with the expression of Ucn 3 in insulin^+^ beta cells (Fig. 7A), but does not label Ucn 3^+^ alpha cells (Fig. 7B), in line with published work that demonstrates that PC1/3 is expressed in human beta but not alpha cells [30]. Staining of grafts of hESC-derived pancreatic endoderm reveals that Ucn 3^+^ beta cells co-express PC1/3 (Fig. 8A, B). A fraction of PC1/3^+^ beta cells does not yet express Ucn 3, which places the appearance of Ucn 3 after that of PC1/3 in the development of hESC-derived beta cells and correlates the onset of Ucn 3 expression with functional maturity. In line with our observations in human islets, Ucn 3 expression in hESC-derived alpha cells does not overlap with PC1/3 expression (Fig. 8C, D).

### Ucn 3 is a maturation marker for human alpha cells

**Figure 9:**
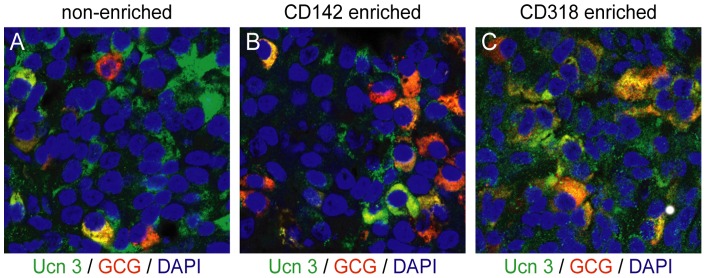
Ucn 3 expression is acquired upon implantation of polyhormonal cells that differentiate primarily into alpha cells. Implantation of hESC-derived pancreatic cultures, either non-enriched (A) or enriched for pancreatic endoderm by CD142 (B) in the epidydimal fat pad for 9 weeks supports the formation of alpha and beta cells that co-express Ucn 3. In contrast, implantation of CD318-enriched polyhormonal cells leads to the formation of predominantly alpha cells that co-express Ucn 3, with no detectable expression of insulin (C).

Our observation that Ucn 3 colocalized with glucagon after implantation of hESC-derived pancreatic cultures into mice raised the possibility that Ucn 3 constitutes a maturation marker for human alpha cells in addition to human beta cells. Therefore, we assessed Ucn 3 expression in grafts of hESC-derived polyhormonal cells that differentiate predominantly into glucagon^+^ mature alpha cells upon implantation *in vivo* [11], [31]. Polyhormonal cells were purified from hESC-derived pancreatic cultures, which do not express Ucn 3 (Fig. 5A–C), on the basis of CD318 expression and grafted in the epidydimal fat pads of mice [11]. Control mice were grafted with CD142-enriched pancreatic endoderm cells or with non-enriched pancreatic cultures [11]. Both non-enriched (Fig. 9A) and CD142-enriched control grafts (Fig. 9B) gave rise to a mixed population of alpha and beta cells, both of which co-express Ucn 3 as previously observed (Fig. 5). In contrast, CD318-enriched cells differentiated predominantly into glucagon^+^ alpha cells (Fig. 9C) in the absence of significant insulin expression (not shown, see [11]). At nine weeks following implantation, these alpha cells had acquired Ucn 3 expression. These findings further support the notion that the acquisition of Ucn 3 expression coincides with alpha cell maturation whether derived by the transition of polyhormonal endocrine precursor cells to glucagon single^+^ cells or by the differentiation of alpha cells from pancreatic progenitors after engraftment in mice.

## Discussion

Ucn 3 was co-discovered by our group a decade ago as the fourth member of the corticotropin-releasing factor family of peptide hormones [32], [33]. We subsequently reported that Ucn 3 is abundantly and exclusively expressed in beta cells of the mouse islet and is required for full glucose- and incretin-stimulated insulin secretion [4], [5]. The great and unmet need for a source of abundant and functional beta cells for a cell-based therapy of type 1 diabetes has initiated many efforts to differentiate beta cells from hESCs or induced pluripotent stem cells (iPSCs). From these studies it has become apparent that insulin expression alone is insufficient to define mature functional beta cells [11], [34], [35], [36]. A recent study reported the onset of Ucn 3 expression in mouse development by immunofluorescence at p6 and suggested that Ucn 3 has utility as a specific maturation marker for hESC-derived beta cells [26]. Our observations indicate that Ucn 3 expression is detectable by immunofluorescence starting at E17.5, over a week earlier than previously reported [26], and is detected in the majority of beta cells by p7. These observations place the onset of Ucn 3 expression past that of insulin to coincide with the early neonatal window that is implicated in the acquisition of functional characteristics of mature beta cells including biphasic glucose-stimulated insulin secretion [37], [38], [39]. Recent findings from the Melton group suggest that early neonatal islets do demonstrate biphasic insulin secretion but at a markedly lower glucose threshold that shifts to a more mature glucose threshold by p15 [26]. Our observations that Ucn 3 is not expressed in *in vitro* generated hESC-derived insulin^+^ cells, but is exclusively induced during differentiation and maturation into Pdx1^+^ or Nkx6.1^+^ beta cells following engraftment, further support the notion that Ucn 3 is a late differentiation marker of insulin^+^ cells in both mice and humans.

Importantly, our observations contrast the findings reported by the Melton group [26], in particular with regards to the distribution of Ucn 3 expression in the human islet. The Melton study reported Ucn 3 expression in human pancreas to be largely restricted to beta cells and absent from alpha cells, and to be upregulated in hESC-derived beta cells following the grafting of hESC-derived progenitors under the kidney capsule of recipient mice [26]. Based on a comprehensive set of experiments that includes immunohistochemistry of human and macaque pancreas and freshly fixed human islets, as well as parallel gene expression profiles of FACS-purified dissociated human islet cells, we conclude that Ucn 3 expression in primates is not restricted to beta cells but extends to alpha cells. The significance of these findings is apparent when assessing the appearance of Ucn 3 expression in hESC-derived mature endocrine cells. In both the human alpha and beta cell lineages, Ucn 3 expression is acquired late in differentiation, following the implantation step currently required for functional maturation [9], [10], [31]. Furthermore, as demonstrated here, Ucn 3 expression in the human beta cell lineage is restricted to mature beta cells that retain expression of Pdx1 and Nkx6.1 and have acquired the PC1/3 expression required to process pro-insulin to insulin. In contrast, Ucn 3 expression in human alpha cells is associated with a notable absence of Pdx1, Nkx6.1 and PC1/3 expression as these factors are absent from mature alpha cells. Moreover, hESC-derived polyhormonal cells that develop primarily into alpha cells in the absence of significant beta cell formation [11], [31], acquire Ucn 3 expression only upon engraftment into the epidydimal fat pad. Collectively, these observations suggest that Ucn 3 in humans is a maturation marker for both the alpha and the beta cell lineages and warrants a more nuanced approach to Ucn 3 and its utility as a marker of beta cell maturation, by assessing insulin and glucagon expression in parallel.

Current protocols for the differentiation of beta cells from hESCs are successful in generating differentiation-competent pancreatic endoderm *in vitro* [7], [9], [10], [11], but the final differentiation steps towards the generation of functional mature beta cells require exposure of these pancreatic endoderm precursors to unknown signals associated with *in vivo* implantation. In light of our observations, induction of Ucn 3 during this differentiation and maturation process cannot necessarily be interpreted as a sign of beta cell maturation, as it could reflect maturation along the alpha cell lineage as well. Notably, following engraftment of hESC-derived Pdx1^+^/Nkx6.1^+^ pancreatic progenitors Blum *et al*. observed Ucn 3 induction in substantially more cells than those that acquired insulin expression [26]. As Pdx1^+^/Nkx6.1^+^ cells have been demonstrated to differentiate into alpha cells (and lose Pdx1 and Nkx6.1 expression in the process) as well as beta cells following engraftment *in vivo* [10], [11], it is possible that some of these Ucn 3^+^ insulin- negative cells are in fact hESC-derived Ucn 3^+^ alpha cells. Similar caution is warranted if Ucn 3 were to be used as a marker for alpha to beta cell transdifferentiation, a process described by several recent studies [16], [40], [41]. While Ucn 3 expression constitutes a potentially useful marker for the acquisition of mature beta cell identity by alpha cells in mouse, the expression of Ucn 3 by human alpha cells precludes its use as a marker for alpha to beta cell transdifferentiation in humans.

In summary, we described here that the expression of Ucn 3, an exclusive beta cell marker in mice [4], is less restricted in primates as it is expressed in primary and hESC-derived human alpha and beta cells. This suggests that human Ucn 3 expression constitutes a general endocrine maturation mark and highlights a potential caveat in the use of Ucn 3 as a maturation marker for hESC-derived beta cells. Nevertheless, we propose that Ucn 3 remains useful to assess hESC-derived beta cell maturation, provided that expression of insulin and glucagon are examined simultaneously to distinguish Ucn 3^+^ mature beta from alpha cells.

## Materials and Methods

### Ethics Statement

Human islets were obtained via the NIDDK-supported Integrated Islet Distribution Program. The Salk Institute for Biological Studies Institutional Review Board declared the human islet material used in this study exempt from IRB review under 45 CFR 46.101 (b) Category (4) on April 16, 2008. All animal experiments were reviewed and approved by Salk Institute Animal Care and Use Committee (IACUC) and performed in compliance with the Animal Welfare Act and the ILAR Guide to the Care and Use of Laboratory Animals. Nonhuman primate samples were obtained from the tissue bank of the Pathology Services of the Salk Institute Animal Resources Department. Tissues were collected as part of other approved experimental studies or during routine diagnostic necropsies. Primates on approved studies were housed, fed and handled according to contemporary standards under the supervision of qualified laboratory animal veterinarians. Every effort was made to alleviate animal discomfort and pain by appropriate and routine use of anesthetic and/or analgesic agents.

### Biological materials

Pancreata or fresh human islets were immersion fixed in 4% paraformaldehyde in potassium phosphate-buffered saline (KPBS) for 3–6 h at 4°C, followed by cryoprotection in 30% sucrose in KPBS overnight. Pancreata were embedded in Tissue-Tek (Sakura Finetek USA, Inc.), frozen on dry ice, and sectioned in 14-μm sections for pancreas and 10-μm sections for human islets on a cryostat and stored at −20°C until use. CyT49 hESCs were maintained and differentiated as described previously [9]. Tissue sections of hESC-derived pancreatic cultures engrafted in the epidydimal fat pads were obtained from ViaCyte and are described in detail elsewhere [9], [11]. Grafts of hESC-derived pancreatic cultures delivered in a TheraCyte macroencapsulation device were provided by ViaCyte (Xie et al., 2012, unpublished data). The diffusion properties of TheraCyte devices have been studied [42]. Briefly, a device loaded with 3×10^6^ cells was inserted subcutaneously into a SCID-Beige mouse under anesthesia. Grafts were harvested at 20 weeks after implantation and subjected to qRT-PCR and Immunohistochemistry analysis.

### Immunohistochemistry

After thawing, slides were washed three times for 5 min in KPBS followed by an overnight incubation at 4°C with primary antibody in KPBS containing 2% normal donkey serum and 0.4% Triton. Slides were washed three times for 5 min in KPBS, incubated for 45 min with secondary antibody and DAPI at 1 μg/ml, followed by three more 5-min washings in KPBS before embedding in Prolong Gold Antifade (Life Technologies). Primary antibodies were guinea pig anti-insulin (Millipore) at 1∶500, mouse anti-insulin (Sigma) at 1∶1000, guinea pig anti-glucagon (Millipore) at 1∶7,000 or Goat anti-glucagon (Santa Cruz) at 1∶50, sheep anti-somatostatin (American Research Products Inc.) at 1∶1,000, mouse anti-Nkx6.1 (BCBC) at 1∶300, mouse anti-PC1/3 (Novus Biologicals) at 1∶50 and guinea pig anti-Pdx1 (generous gift of Dr. Chris Wright) at 1∶1000. Rabbit anti-human Ucn 3 (#6570; 1∶1,000) and rabbit anti-rat Ucn 1 (#5779; 1∶1,000) were generated in house. Secondary antibodies were Alexa Fluor 488-conjugated donkey anti-rabbit or donkey anti-goat (Molecular Probes), Alexa Fluor 488-conjugated goat anti-mouse IgG F(ab')_2_, Cy3-conjugated donkey anti-guinea pig, Dylight 549-conjugated donkey anti-rabbit, DyLight 649-conjugated donkey anti-sheep or donkey anti-guinea pig, Cy5-conjugated donkey anti-goat (all Jackson ImmunoResearch Laboratories Inc., West Grove, PA), at 1∶600. Mouse primary antibodies were used in conjunction with the MOM blocking kit (Vector Labs) per the manufacturer's instructions. Staining for Nkx6.1 required antigen retrieval in 10 mM sodium citrate by microwave. Slides were imaged using Zeiss LSM710 or 780 confocal microscopes (Zeiss) at the Waitt Advanced Biophotonics Center Core Facility of the Salk Institute for Biological Sciences.

### FACS sorting

Human islets (20,000 IEQs) were dissociated in 0.25% trypsin-EDTA solution (Life Technologies) for 5 min at 37°C aided by gentle mechanical dissociation by pipetting. Cell surface labeling was carried out according to [25]. In brief, dissociated primary islet cells were washed in CMRL with 2% FBS, passed through a 40 micron cell strainer and resuspended at 5×10^6^ cells/ml. Cells were incubated with HIC1-2B4 and HIC3-2D12 mouse hybridoma supernatant at 1∶50 for 30 minutes on ice, washed in CMRL (serum-free) followed by incubation with Alexa Fluor 488-conjugated goat anti-mouse IgG F(ab')_2_ or phycoerythrin-conjugated goat anti-mouse IgM F(ab')_2_ secondary antibodies respectively (both 1∶200 in serum-free CMRL; Jackson ImmunoResearch Laboratories Inc., West Grove, PA). Cells were sorted on a FACS Aria with an added 561 nm laser line (Becton-Dickinson, Franklin Lakes, NJ) and 20,000 to 100,000 cells were collected in Trizol for each population for gene expression analysis. Dissociated mouse islets from *MIP-GFP* transgenic animals were sorted as previously described [20]. Sorting of hESC-derived pancreatic cultures with CD200 to isolate polyhormonal endocrine cells and with CD142 to isolate pancreatic endoderm cells was performed as previously described [11].

### Gene expression

RNA was isolated by adding 200 μl chloroform per ml of Trizol, followed by vigorous mixing and 15 min, 12,000 rcf spin at 4°C. The aqueous phase was transferred to a fresh tube and mixed with 500 μl isopropanol per ml of Trizol prior to application on an RNeasy micro spin column (Qiagen). After spinning the sample through, the column was washed with 350 μl wash buffer RW1, prior to on column DNAse treatment per the manufacturers instructions. The column was washed with 350 μl of wash buffer PE and spun extensively to avoid any carry over of ethanol, prior to elution of RNA in 30 μl of RNAse-free water. Reverse transcription was conducted with the cDNA archive kit (Applied Biosystems) and gene expression was assessed by qRT-PCR using SYBR chemistry on a Lightcycler 480 platform (Roche Diagnostics) with primers listed in Table 1.

**Table 1:** Primer information.

gene	Primer	sequence 5′ ⇒ 3′	expected amplicon
HPRT	qhHPRT.fwu1	GACCAGTCAACAGGGGACAT	95 bp
	qhHPRT.rvu1	GTGTCAATTATATCTTCCACAATCAAG	
TATA-box	qTBP.fw1	TGTGCACAGGAGCCAAGAGT	51 bp
binding protein	qTBP.rv1	ATTTTCTTGCTGCCAGTCTGG	
insulin	qhINS.fw1	GCAGCCTTTGTGAACCAACA	71 bp
	qhINS.rv1	CGTTCCCCGCACACTAGGTA	
glucagon	qhGCG.fw1	GCTGCCAAGGAATTCATTGC	94 bp
	qhGCG.rv1	GTCTGCGGCCAAGTTCTTCA	
somatostatin	qhSST.fw1	CTGTGTCACCGGCGCTC	123 bp
	qhSST.rv1	TTGGGTTCAGACAGCAGCTCT	
Ucn 3	qhUcn3.fw1	GAGGGAAGTCCACTCTCGGG	137 bp
	qhUcn3.rv1	TGTTGAGGCAGCTGAAGATGG	
